# Identification of tumor epithelium and stroma in tissue microarrays using texture analysis

**DOI:** 10.1186/1746-1596-7-22

**Published:** 2012-03-02

**Authors:** Nina Linder, Juho Konsti, Riku Turkki, Esa Rahtu, Mikael Lundin, Stig Nordling, Caj Haglund, Timo Ahonen, Matti Pietikäinen, Johan Lundin

**Affiliations:** 1 Institute for Molecular Medicine Finland (FIMM), P.O. Box 20, FI-00014 University of Helsinki, Helsinki, Finland; 2 Machine Vision Group, Department of Electrical and Information Engineering, University of Oulu, P.O. Box 4500, FI-90014 Oulu, Finland; 3 Department of General Surgery, Helsinki University Central Hospital, PO Box 340, Haartmaninkatu 4, Helsinki, 00290 HUS, Finland; 4 Department of Pathology, Haartman Institute, University of Helsinki, Haartmaninkatu 3, P.O. Box 21, FI-00014 University of Helsinki, Helsinki, Finland; 5 Department of Pathology, Helsinki University Central Hospital, Haartmaninkatu 3, Helsinki, FI-00014, Finland; 6 Visual Computing and Ubiquitous Imaging Research Team, Nokia Research Center, Palo Alto, CA, USA; 7 Division of Global Health/IHCAR, Karolinska Institutet, Nobels väg 9, SE-171 77 Stockholm, Sweden

**Keywords:** Image analysis, Texture classification, Pattern recognition, Stroma, Epithelium, Local binary patterns, Haralick, Gabor, Support vector machine

## Abstract

**Background:**

The aim of the study was to assess whether texture analysis is feasible for automated identification of epithelium and stroma in digitized tumor tissue microarrays (TMAs). Texture analysis based on local binary patterns (LBP) has previously been used successfully in applications such as face recognition and industrial machine vision. TMAs with tissue samples from 643 patients with colorectal cancer were digitized using a whole slide scanner and areas representing epithelium and stroma were annotated in the images. Well-defined images of epithelium (n = 41) and stroma (n = 39) were used for training a support vector machine (SVM) classifier with LBP texture features and a contrast measure C (LBP/C) as input. We optimized the classifier on a validation set (n = 576) and then assessed its performance on an independent test set of images (n = 720). Finally, the performance of the LBP/C classifier was evaluated against classifiers based on Haralick texture features and Gabor filtered images.

**Results:**

The proposed approach using LPB/C texture features was able to correctly differentiate epithelium from stroma according to texture: the agreement between the classifier and the human observer was 97 per cent (kappa value = 0.934, *P *< 0.0001) and the accuracy (area under the ROC curve) of the LBP/C classifier was 0.995 (CI95% 0.991-0.998). The accuracy of the corresponding classifiers based on Haralick features and Gabor-filter images were 0.976 and 0.981 respectively.

**Conclusions:**

The method illustrates the capability of automated segmentation of epithelial and stromal tissue in TMAs based on texture features and an SVM classifier. Applications include tissue specific assessment of gene and protein expression, as well as computerized analysis of the tumor microenvironment.

**Virtual slides:**

The virtual slide(s) for this article can be found here: http://www.diagnosticpathology.diagnomx.eu/vs/4123422336534537

## Background

Tissue microarrays (TMAs) are the standard for high-throughput analysis of diagnostic, prognostic and predictive tissue biomarkers [1] and for rapid validation of molecular expression patterns in large-scale tissue materials [2]. However, the extensive tissue sample series included in TMAs give rise to bottlenecks in the manual microscopy-based evaluation of immunostaining and *in situ *hybridization results.

Computer-assisted automated quantification of immunohistochemical protein staining has previously been shown to be feasible in TMAs [3-6] and resulted in higher reproducibility compared to human-based judgment [7]. Tissue compartment specific quantification of molecular expression patterns remains a challenge for computer-assisted methods. A skilled human observer easily segments the tissue into compartments and can report immunohistochemical staining in tumor cells and stroma separately. Computerized segmentation of morphology, on the other hand, is currently largely dependent on special dyes e.g. fluorescent tags that can separate tumor cells from stroma [7]. Since the spatial location (in tumor cells *vs*. stroma) of protein expression can be of biological and clinical relevance [8-10], more efficient methods for computerized segmentation are needed.

In this study we have focused on the analysis of image texture to improve segmentation of tissue into specific tissue compartments. Texture analysis has achieved high accuracy in a series of pattern classification problems [11]. These techniques, stemming from pattern recognition and machine learning, have improved during the last years both due to methodological advances, and because of extended computational capacities. Texture is a fundamental property of surfaces, including sections of tissue. Various texture analysis methods have been developed. For example, statistical methods based on co-occurrence matrices, signal-processing methods based on local linear transforms, multichannel Gabor filtering or wavelets, and model-based methods such as Markov random fields or fractals [11]. Only a few studies have been published regarding automated segmentation of tissue images and are either approaches taking advantage of color space methods [12,13], texture analysis [14-16] or other morphology-based algorithms [17].

A texture analysis method that has been efficient in a variety of pattern classification tasks is based on local binary patterns (LBP) [11,18,19]. Part of the success of the LBP is due to the rotation and gray scale invariance. We hypothesized that the rotation invariance would be important in the analysis of microscopy images of tissue specimen where control of the spatial sample orientation is difficult or impossible to attain. Equally, the gray scale invariance could compensate for variation in sample staining (e.g. due to differences in color and sample thickness), illumination conditions and camera settings.

For the purpose of evaluating a texture analysis method for computerized segmentation of tissue samples, we here report the performance of an LBP algorithm combined with a contrast measure (LBP/C) to discriminate epithelial regions from stroma in a series of digitized colorectal cancer TMAs. The performance of the LBP/C algorithm was evaluated against corresponding algorithms based on Haralick textures [20] and Gabor filtered images [21].

## Methods

### Patient series

The study is based on tissue samples from a series of 643 consecutive patients who underwent surgery for histologically verified colorectal cancer at the Helsinki University Central Hospital in 1989 to 1998. The clinico-pathological characteristics of the patients in this series have been described in detail previously [22].

Permission to use clinical data and formalin-fixed, paraffin-embedded tissues for research purposes was provided by the National Authority for Medical Affairs, Finland (Permission# 3990/04/046/07). With reference to the large number of patients studied and because a considerable number of the persons from whom the samples are derived were not alive at the time when the study was started, the authorities granted permission to use tissue samples without individual patient consent. Additionally, the study was approved by the local Ethics Committee and complies with the Declaration of Helsinki (Permission# HUS 226/E6/06).

### Tissue samples and preparation of tumor tissue microarrays

Representative tumor regions in routinely fixed paraffin-embedded samples were defined from H&E-stained sections and marked. Donor tissue blocks were sampled and three cores punched from each donor block and transferred to the tissue microarray blocks. From the 643 tumor samples, 27 tissue array blocks were prepared, each containing 10-180 tumor samples. Eight tissue arrays were selected for the study, representing one core per tumor. Sections of 4 μm were cut from the TMAs and further processed for immunostaning with epidermal growth factor receptor (EGFR) antibody. Of note is that this particular EGFR immunostaining is not relevant with regard to the objectives of the current study. For immunohistochemistry of EGFR a Lab Vision Autostainer TM 480 (LabVision, Fremont, CA) was used. Deparaffinised formalin-fixed, paraffin-embedded tissue sections were heated in the pretreatment module of the autostainer in Tris-HCl pH 8.5 buffer (for 20 minutes at 98°C). For inactivation of endogenous peroxidises, the sections were incubated (for 5 minutes) in Peroxidase Block Solution (DAKO, Carpinteria CA) and incubated for 30 minutes with the primary antibody NCL-EGFR (Novo Castra, Newcastle upon Tyne, UK), diluted 1:10. The sections were then reacted (for 30 minutes) using the Advance HRP detection system (DAKO, Carpinteria CA). The reaction products were revealed with the brown colored chromogen diamino-benzidine (DAB) and finally the sections were counterstained with haematoxylin (for 1 minute).

### Digitization of stained tissue microarray slides

The tissue micorarray array slides were digitized with an automated whole slide scanner (Mirax Scan, Zeiss, Göttingen, Germany), using a 20 × objective (numerical aperture 0.75) and a Sony DFW-X710 camera (Sony Corporation, Tokyo, Japan) equipped with a 1/3" type 1034 × 779 pixel CCD sensor. The pixel resolution was 0.26 μm. Images were compressed to a wavelet file format (Enhanced Compressed Wavelet, ECW, ER Mapper, Erdas Inc, Atlanta, Georgia) with a conservative compression ratio of 1:5.

### The virtual microscopy platform

The compressed virtual slides were uploaded to our web server (http://www.webmicroscope.net) running image server software (Image Web Server, Erdas Inc, Atlanta, Georgia). Virtual slides on the server can be viewed and processed with image analysis algorithms (i.e. ImageJ and MATLAB) using a standard web browser interface. The user is able to navigate into an area-of-interest in a whole slide sample or TMA, and store the current view as a region-of-interest that subsequently can be processed by image analysis. The image algorithms in the current study were run on a server equipped with a 3.33 GHz Intel Core i7 processor and six cores, and 24,0 GB RAM.

### Annotation of representative tissue regions and image data set

For training of the algorithm representative epithelial (n = 41) and stromal (n = 39) regions-of-interest were defined in the digitized TMA slides. The training set images were only used for training. A separate validation set (n = 576) was defined for optimization of the algorithms and consisted of 360 images representing epithelium and 216 images representing stroma. Finally, a test set (n = 720) was defined for assessment of classifier accuracy and consisted of 425 images representing epithelium and 295 images representing stroma.

The images used for training, validation and testing are stored in a database and available at http://fimm.webmicroscope.net/supplements/epistroma. Image annotation was carried out by one of the researchers (N.L.) and verified by a pathologist (S.N.).

The dimensions of the annotated areas varied between 93-2372 in pixel width and 94-2373 in pixel height. Magnification was constant i.e. images were always of the same pixel resolution although the annotated area was variable.

#### Preprocessing

To extract the texture features, the tissue sample images are first scaled, then converted to grayscale and finally possible background area is removed.

In the current study, images were scaled by a constant of 0.5. The grayscale conversion is performed by computing a weighted sum of the R, G and B components of the color image: 0.2989 * R + 0.5870 * G + 0.1140 * B.

Possible background was removed by creating a binary mask in which the foreground tissue pixels were marked by ones and the background pixels by zeros. In bright field microscope images, the background pixels have high luminance values. These bright areas were removed from the gray-scale image by a threshold value of 240. Structures in the resulting binary mask were smoothed morphologically by closing and eroding the binary image [23]. The binary mask was used later to prune areas scarce of tissue i.e., the background.

#### Feature extraction

The downscaled images were divided into blocks and the classification was performed by processing the blocks independently. The blocks were defined by sliding a square of 80 × 80 pixel window through the image. The window was moved row by row from the upper left corner to the lower right by 40 pixels at the time, thus creating a 50% overlap. If the area of a background binary mask that corresponds to the area of a block contained 50% or more tissue, the particular block was processed, if not, the block was considered as background, and it was not further processed.

### Texture features

#### Local binary patterns

**Figure 1:**
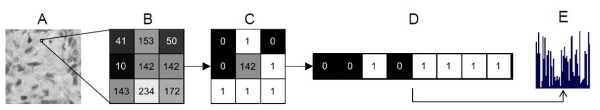
**Local binary patterns (LBP) are defined for an image (A) based on its grayscale values**. For every 3 × 3 pixel neighborhood (**B**) within the image, an LBP code is generated by thresholding the surrounding pixels using the value of the central pixel (**C**-**D**). A histogram (**E**) of the all LBP codes within the analyzed image is formed to represent texture properties of the image

The local binary pattern operator (LBP) compares each pixel in an image to *P *pixels in a circular neighborhood with radius *R*. The intensity value of the central pixel is used to threshold the surrounding pixels forming a binary code (Figure 1). The pixels, which have a value less than the value of the central pixel, are set to 0, and the pixels that have a larger or equal value are set to 1. The binary code is interpreted as a base-2 number i.e. its digits are weighted by the powers of two to form the analogous base-10 number, for instance a binary code 10011011_2 _represents an LBP code number 155_10_. The original LBP [19] was defined in a rectangular 3 × 3 pixel neighborhood (*P *= 8, *R *= 1) for gray-scale images, but the radius of the operator can be extended to include pixel neighborhoods farther from the central pixel (e.g. *P *= 16, *R *= 2).

Invariance to rotation can be achieved using minimized uniform patterns [18]. Patterns, which have at most two transitions on a circular ring from 1 to 0 or vice versa are called uniform. The uniform patterns are minimized by bit shifting the LBP code to a position where it reaches its minimum, for instance uniform patterns like 00011110, 11000011 and 11110000 are shifted to 00001111 = 15. When uniform patterns are used, all the non-uniform patterns are mapped to one LBP code. This restricts the number of possible LBP codes to *P *+ 2.

By definition the LBP discards contrast, while the LBP feature is strongly characterized by its capability to detect variations in the structure of the texture pattern. To capture also the contrast information, i.e. the strength of the texture patterns, the LBP was combined with a rotation invariant local variance (VAR) [18]. As for the LBP, the VAR is formulated in a circular neighborhood, often with the same radius *R *and sample points *P *as the LBP. Essentially the VAR represents the variance of the gray values of the surrounding pixels i.e., the sample points.

The joint distribution of the above-described operators is used to merge the contrast (C) with the LBP pattern, i.e. LBP/C. To determine the joint distribution, the output VAR is quantized to eight *Q *levels. The quantization is performed by computing VAR for a set of training images and then dividing the distribution of VAR values into *Q *levels, each having an equal number of pixels. This restricts the size of the joint distribution to (*P *+ 2) × *Q *discrete bins. MATLAB implementations for LBP and VAR operators presented here are available at http://www.cse.oulu.fi/MVG/Downloads. For each block, a numerical representation of its texture was computed by using two discrete joint distributions: $LBP_{8,1}^{riu2}\;+\;VAR_{8,1}$ and $LBP_{16,2}^{riu2}\;+\;VAR_{16,2}$. The histograms were concatenated to one (8 + 2) × 8 + (16 + 2) × 8 = 224 bins long feature vector. The Euclidean norm of the feature vector was normalized to one.

#### Haralick textures features

The Haralick texture descriptor is a metric representation that is dependent on the spatial gray level dependence matrices, i.e. co-occurrence matrix *C*_Δ*x*,Δ*y*_∈ *R^M×M^*, where Δ*x*,Δ*y *defines the offset used to construct the matrix. In a certain image with *M *gray levels, the spatial gray level dependence matrix at angle *θ *is a matrix of size *M *× *M*. In the matrix, each element is a sum of the total number of pairs of gray levels at the predefined offset over the whole image. In the current study, image gray scale values were linearly quantized to 8 levels; which define the size of the co-occurrence matrix *R*^8 × 8^. Three symmetrical co-occurrence matrices with offset pairs (0,1), (1,1) and (1,0) were used to describe second-order statistics. The following metrics were computed from the matrices and used as input for the classifier; autocorrelation, contrast, correlation, cluster prominence, cluster shade, dissimilarity, energy, entropy, homogeneity, maximum probability, sum of squares, sum average, sum variance, sum entropy, difference variance, difference entropy, information measure of correlation 1, information measure of correlation 2, inverse difference normalized and inverse difference moment normalized [20,24,25].

#### Gabor filters

The Gabor filters are a group of Gabor wavelets, a filter bank, which may be designed for different dilations and rotations. For texture analysis purposes the input image is filtered with the filter bank and then a set of descriptors are computed from the resulting output images. Gabor functions have properties that make them suitable for texture applications, i.e. tunable bandwidths, the option to be defined to operate over a range of spatial frequency channels, and acting upon the vagueness principle in two dimensions [21].

In the current study, Gabor features were computed from the filter bank defined by the orientation parameter *θ *= *nπ*6, n∈0,...,5 and scale parameter *s *∈ 0,....,3. For each parameter combination a unique Gabor transformation was defined, and for classification purposes the mean and the standard deviation of the magnitude of the transformation coefficients were used. The above-mentioned parameter settings yield component feature vector that was used as input for the classifier.

#### Classification

A linear support vector machine (SVM) was used to classify the image blocks extracted from the input images. The SVM classifies data based on a model that it has learned from a given training set. LBP/C, Haralick and Gabor features and their class labels were used to train the SVM classifier model. Then the trained classifier was optimized with images from the validation set and finally tested with the independent test set images. The model describes the hyperplane that separates the classes of the training set with the largest possible margin. A library for large linear classification (LIBLINEAR) [26] was used to implement a linear capacity constant SVM (C-SVM).

#### The algorithm output

**Figure 2:**
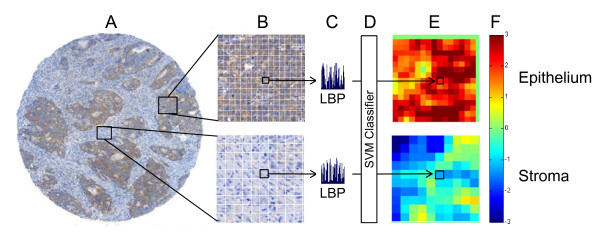
**Principle of image annotation, block-based feature extraction and classification**. Areas representative of pure tumor epithelium and stroma were identified in the digitized tissue microarray spots (**A**) and then split into blocks of size 80 × 80 pixels (**B**). A local binary pattern (LBP/C) operator was applied to the blocks and block-specific LBP histograms generated (**C**). The block histograms are then used as input to a support vector machine (SVM) classifier (**D**), which assigns a tissue category (epithelium or stroma) score to the block. The SVM score for each block is pseudo colored to visualize the output (**E**), and the average block score is taken to represent the predicted class of an image (**F**)

The analyzed images differed in size (pixel dimensions) and therefore contained a varying number of blocks that the SVM classified (Figure 2). The average SVM score of all blocks in an image defined to which class the test image was assigned, i.e. epithelium or stroma. The sign of the classification score indicates on which side of the decision hyperplane a feature vector lays, i.e. it represents the predicted class. Additionally, the absolute value of the classification score is the distance between the feature vector and the decision hyperplane. The points near the hyperplane in the feature space are more likely erroneous than the ones that are further from it; hence the decision value is a measure of the certainty of the prediction. Images with an SVM score lower than -1 or higher than 1 were therefore regarded as strong candidates for the respective classes, whereas those closer to zero (SVM score between -1 and 1) were considered as weak candidates. The decision value threshold for the classification into the stroma and epithelium categories was set to zero.

**Figure 3:**
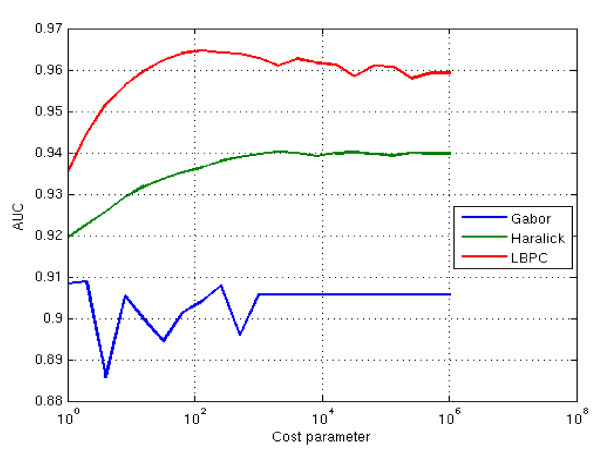
**Performance results for LBP/C, Haralick and Gabor texture descriptors on the validation set (colorectal cancer images; n = 576)**. A linear classifier was optimized for each of the descriptors by computing the accuracy, AUC (area under the ROC curve) over a set of C values (Cost parameter) growing in exponential sequence C = 2^0^,..., 2^20^. The AUC was computed on block level. The selected C values based on the validation tests were: LBP/C; C = 300, Haralick features; C = 2048, and Gabor filters; C = 2

**Figure 4:**
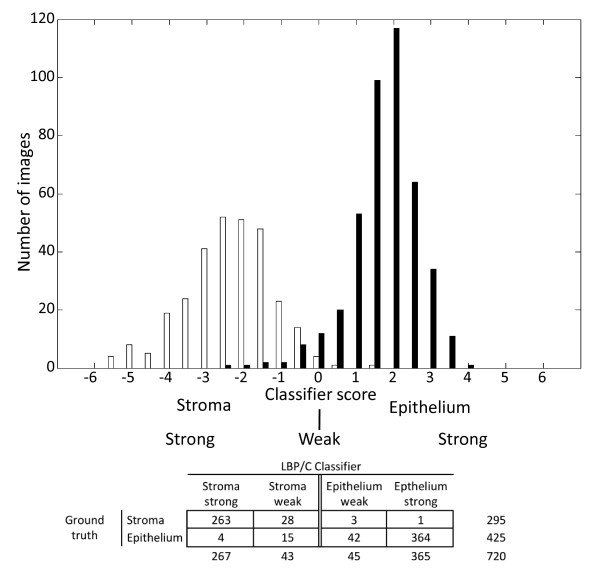
**Contingency table for discrimination of colorectal cancer stroma and epithelium images in the test set (colorectal cancer images; n = 720) using the local binary pattern (LBP/C) classifier**. The value of the score generated by the classifier defines to which class the test image is assigned, i.e. strong or weak epithelium (black bars) or stroma (white bars)

In the results images the pixels that correspond to the pixels of the block in the original image are pseudo-colored according to the decision value of the particular block (Figures 2, 3, and 4). For the overlapping areas average values of the overlapping decision values are computed. A heat map (color map) that maps large positive values to dark-red (most likely to be epithelium) and large negatives to dark-blue (most likely to be stroma) was used to generate the pseudo-colored segmentation image (Figures 2, 3, and 4). The colors between the extremes change from light blue and turquoise to light green, and from light green to yellow and orange. Light green color represents zero or almost zero values, which corresponds to image blocks whose correct class the algorithm is least certain of.

### Statistical methods

The accuracy of the classifier was evaluated with regard to discrimination by calculation of the area under the receiver operating characteristic curve (AUC). The AUC can be interpreted as the probability that for any randomly chosen pair of tissue image samples, one that represents epithelium and the other stroma, the classifier will assign a higher score to the former. An AUC of 0.5 indicates a random classifier and AUC 1.0 a perfect classifier. The agreement between the visual and automated methods in the assessment of tissue type was estimated by percent-agreement and kappa-statistics.

## Results

The LBP/C, Haralick and Gabor texture classifiers were optimized on the validation set of 576 colorectal cancer microscopy images (http://fimm.webmicroscope.net/supplements/epistroma). Of these images, 360 represented epithelium and 216 stroma. Optimization was done by computing the accuracy (area under the ROC curve) over a set of cost parameter values, C for the linear support vector machine classifier for each of the LBP/C-, Haralick- and Gabor descriptors. The selected C values based on the validation tests were: LBP/C; C = 300, Haralick; C = 2048, and Gabor; C = 2 (Figure 3).

**Figure 5:**
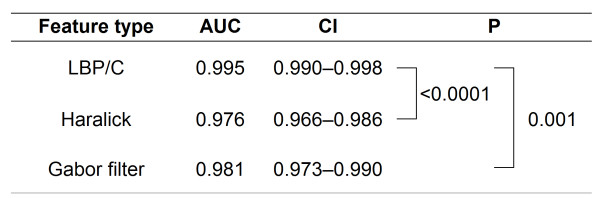
**Summary of feature types and accuracy (area under the ROC curve) for each feature type in the test set (colorectal cancer images; n = 720) respectively**. CI = confidence interval.

For testing of the LBP/C, Haralick and Gabor classifiers, 720 colorectal cancer images (http://fimm.webmicroscope.net/supplements/epistroma) were used. The images used for testing were different from those used for optimization of the different texture feature sets. Of the images used for testing, 425 represented epithelium and 295 stroma. The accuracy (area under the ROC curve) of the LBP/C texture classifier was 0.995 (CI 95% 0.991-0.998), for Haralick features 0.976 (CI 95% 0.966-0-986) and for Gabor filtered images 0.981 (CI95% 0.973-0.990) for assigning the correct class to the test images. A significant difference between the accuracy for the LBP/C classifier and the Haralick features as well as between the LBP/C classifier and Gabor filtered images were observed (Figure 5). The running time for analyzing the test set (n = 720) was 99 seconds using the LBP/C algorithm, 47 seconds using Haralick features, and 145 seconds Gabor filtering.

The highest accuracy for assigning the accurate class to the test images was achieved by the LBP/C classifier; therefore these results were analyzed in more detail. The sensitivity of the LBP/C classifier for correctly identifying the stroma images in the test set was 99% (CI95% 98%-99%) and the specificity was 94% (CI95% 92%-95%). The agreement percentage between the texture classifier and the human observer was 97% (kappa value = 0.93, *P *< 0.0001) (Table 1).

**Table 1:** Contingency table for discrimination of colorectal cancer stroma and epithelium, in the test set (colorectal cancer images; n = 720) using the local binary pattern (LBP) texture algorithm.

		Automated classifier		
		**Stroma**	**Epithelium**	
Human observer	Stroma	291	4	295
	Epithelium	19	406	425
		310	410	720

Total correct classification was 97 per cent (kappa value = 0.93, *P *< 0.0001)

**Figure 6:**
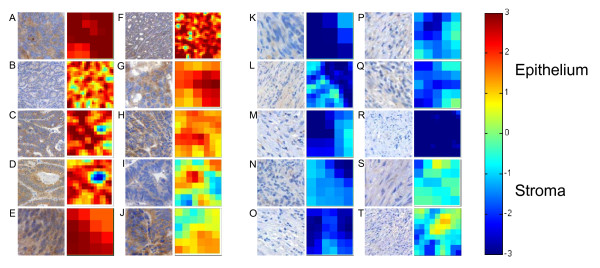
**Example images of epithelial and stromal tissue in the test set (colorectal cancer images; n = 720)**. A-H represents examples of histological images that have been strongly- and I-J images that have been weakly classified as epithelium by the local binary pattern classifier. K-R represents examples of tissues that have been strongly assigned into stroma, and S-T images that have been weakly classified as stroma

In the test set using LBP/C features, the average SVM score in the epithelium images was 1.73 (SD 0.89, range -2.3 to 3.8) and in the stroma images -2.37 (SD 1.16, range -5.6 to 1.3) (Figure 4). Of the 425 epithelium images, 364 were strongly assigned to the correct category and 42 weakly (Figures 4 and 6A-E). Of the 295 stroma images, 263 were strongly and 28 weakly assigned to the correct class (Figures 4 and 6F-J). The algorithm incorrectly classified a total of 23 images, i.e. 4 images were wrongly classified as epithelium and 19 images were wrongly classified as stroma.

**Figure 7:**
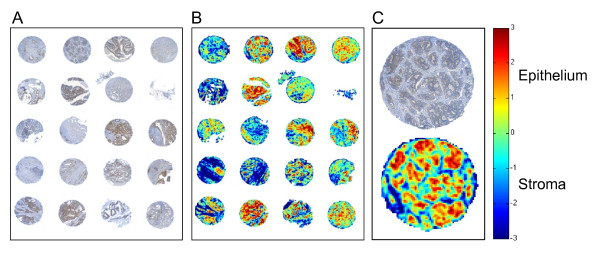
**A part of a digitized colorectal cancer tissue microarray (TMA) immunostained with epidermal growth factor receptor (EGFR) antibody (A) and the same TMA as processed by the local binary pattern (LBP/C) classifier (B)**. One representative tissue spot and its corresponding LBP/C result image. The bar on the right shows the heat map for the LBP/C classifier score values

To visualize the result of the LBP/C texture analysis method when processing larger areas of tissue we analyzed a whole TMA with 73 colorectal tumor tissue spots (Figure 7, accessible at http://fimm.webmicroscope.net/supplements/epistroma).

## Discussion

In the present study we evaluated a texture analysis approach using LBP texture features in combination with a machine learning method to identify tissue types in a large series of digitized colorectal cancer TMAs. Segmentation of tumor tissue into epithelium and stroma facilitates automated assessment of protein expression within the respective tissue compartments. Protein expression quantification can be performed as a sequential process in which a primary algorithm performs the segmentation and a secondary algorithm calculates the area and intensity of an immunohistochemical staining. Computerized tissue type-specific interpretation of immunohistochemical staining has the potential to produce more reliable and reproducible results as compared to visual quantification methods by a human observer [27]. In addition, an algorithm that identifies tumor epithelium could be utilized for the purpose of identifying regions of interest to be punched from the donor block in the process of TMA construction or for laser capture micro dissection of specific cells of interest [28].

The tumor epithelium can exhibit a range of textures, from an appearance close to the normal tissue in well-differentiated cancer to the lack of organizational features in poorly differentiated tumors. Epithelial tissue texture is different from stromal texture which is organized in specific directions and is loosely arranged [29]. Several powerful pattern recognition methods have emerged during the last few years, especially within texture classification [11]. As a rule many of these techniques assume that the textures are uniformly presented and captured in the same orientation. In the analysis of tumor tissue, samples are cut in various planes and positioned at different angles on slides for analysis. Thus, uniform orientation is not possible to achieve and analysis of tissue texture should be invariant to orientation. Also, the algorithm should be robust with regard to variations in image contrasts due to tissue processing and factors related to image acquisition.

The LBP operator is a rotation and grayscale invariant texture descriptor and is therefore interesting in the context of tissue texture analysis. LBP has been successfully used in various applications. For example, the LBP algorithm is used for face recognition [30] and other applications within biometrics, including iris recognition [31] and fingerprint identification [32]. The LBP operator has been proven to be highly discriminative and its key advantages are computational efficiency and invariance to monotonic gray level changes [19].

Texture-based algorithms for classification of tumor tissue have, to some extent, been previously studied, but generally included only small specimen series. In one study, image texture analysis was used for mapping dysplastic fields in colorectal tissue [33]. Another study showed that identification of normal *vs*. abnormal prostatic tissue components in large-scale histological scenes was feasible using Haralick's co-occurrence texture features [16]. For classifying breast histology images, texture-based operators using supervised learning have been employed [34,35]. In a recent publication, a wavelet-based, multiscale framework for texture-based color image segmentation was used to differentiate various tissue compartments in ovarian carcinoma. In that study an average of 71.5% of pixels were assigned to the correct class by the algorithm i.e. five tissue types manually annotated in the images by the human observer [36]. A direct comparison with our results is not feasible, since we focused on the discrimination between two tissue types. Also, in the current study we analysed the accuracy on an image-block level in comparison to studies that report pixel-level results. We argued that obtaining a ground truth with regard the tissue categories (stroma and epithelium) as defined by a human observer on a pixel-level would not be possible without substantial inter-observer variability.

In a few earlier reports, the LBP algorithm has been adapted for tissue classification. In a previous report that compared different histogram-based feature sets for tissue images, the LBP obtained the highest classification accuracy [37]. Another approach using LBP was employed to determine tissue as either stroma-rich or stroma-poor from digitized whole-slide neuroblastoma slides. The approach was tested on 43 whole-slide samples and provided an overall classification accuracy of 88% [15]. The LBP/C algorithm described here discriminates between epithelium and stroma with a higher accuracy (99%) than the method presented by Sertel et al. This may be partly due to differences in the tissue architecture in neuroblastoma as compared to the morphology of the stroma in colorectal cancer tissue. In addition, differing LBP parameters, classifier selections (SVM *vs. k*-nearest neighbor), and incorporation of the contrast information might explain part of the difference in accuracy. In another study LBP features were used for classification of sub-cellular protein localization and also, the algorithm has been applied on pap smears to classify cervix cells as either normal or abnormal [38,39].

In the present study the accuracy of the LBP/C texture classifier for assigning the correct histological class was significantly higher with the LBP/C operator as compared to Haralick features and Gabor filters. The LBP operator can be seen as a unifying method to the traditionally divergent statistical and structural models of texture analysis. The rotation invariance and tolerance against illumination changes of the LBP operator may be factors that have an impact on the outcome in our setting. Regarding Gabor filters, it has been suggested they have a tendency to over-represent low frequency components and under-represent higher-frequency components and thus may not always be suitable for texture analysis of natural images [40]. Since the discriminative accuracy of all three descriptors was excellent (AUC > 0.95) no firm conclusions on the superiority of one single approach can be drawn and performance results may vary according to the analyzed tissue type.

As mentioned previously, the current method is based on image blocks, with a size of approximately 40 micrometers. Thin rows of tumor cell or non-stromal cells interspersed with stroma might therefore be wrongly classified as stroma. Future studies are needed to assess resolution requirements for segmentation of specific tissue types or disease states (e.g. infiltrating inflammatory cells).

We used colorectal cancer as a model to test the ability of the texture algorithm to differentiate the two histological tissue types. Whether our results will be applicable to other cancer types than colorectal cancer, needs to be explored in further research. In this study we analyzed a series of tissue samples immunoassayed for analysis of the EGFR protein and visualized by the DAB chromogen. It cannot be ruled out that immunohistochemical staining process influenced the results, although the algorithm should be invariant to color/image intensity. Also, the methods used for antigen retrieval may modify tissue architecture and thus the texture of the tissue. The reason for us to choose the EGFR protein staining was that the staining was of good quality, i.e. there was only little cross reactivity between the epithelial and stromal compartments. We analyzed immunohistochemically stained tissue sections and not haematoxylin-eosin stained tissue, because our aim was to test the performance of the algorithm on samples prepared for tissue protein expression analysis.

In future studies, it will be of interest to apply texture analysis on other cancers, e.g. breast- and prostate tumor samples. A computerized segmentation into tumor epithelium and stroma would be of relevance in studies regarding the tumor microenvironment, especially when applied to large series on digitized whole slides samples. Stromal cells and their roles in cancer prognosis [8] and response-prediction [9] have been increasingly recognized. It has been proposed that induction or loss of certain proteins in the stroma may be critical in promoting the metastatic phenotype in cancers [10].

In addition to the segmentation of tumor tissue to specific compartments described in this study, texture classifiers for cancer tissue in combination with clinical and bio-molecular data may act as prognostic markers [41]. By probing large sample areas and thousands of tissue specimens, previously undiscovered texture patterns for cancer with clinical and prognostic relevance could potentially be identified. Texture-based algorithms also have the potential to be used for more general tissue segmentation and image quality assessment in whole-slide images [42,43]. Texture features combined with color information might be of interest and is currently a highly investigated topic in computer vision [44].

## Conclusions

In this study we have adopted texture-based methods for classification of epithelium and stroma in a large set of human colorectal cancer. The accuracy of classifiers based on LBP/C, Haralick features and Gabor filters, in discriminating between the two histological tissue types was consistently high. Together with the rapid development of large-scale image processing methods, computer vision based texture classifiers are excellent candidates for automated quantification of tissue-specific proteins in tumor samples and to identify regions of interest for TMA construction in high throughput settings.

## Competing interests

The authors declare that they have no competing interests.

## Authors' contributions

JL designed the study and was in charge of it. NL coordinated the study and identified the regions of interest from the colorectal cancer tissue microarrays and SN confirmed the accuracy. ML wrote the code for the virtual microscopy platform and participated in data acquisition and analysis. JK, RT, ER, TA and MP designed, and RT performed, the computational experiments. CH devised the colorectal cancer patient series. NL, JL and JK drafted the manuscript and RT and ER contributed to improving the draft of the manuscript. All authors read and approved the final manuscript.
